# The Spirit of Imitation in Medicine

**Published:** 1886-10

**Authors:** 


### The Spirit of Imitation in Medicine.

No professional man at all aquainted with the trend of medical opinion in the United States can have failed to note the astonishing subservience of that opinion to all that comes to us from across the Atlantic. This is especially true as regards everything medical emanating from Germany. Let some tormented spirit discover a new "microbe;" let another victim of histological restlessness pounce upon yet other "microbes" whose predatory instincts lead them to eat up "microbe" No. 1, and straightway we fall prostrate before the prestigitator who has afforded us such unique entertainment. There is no harm in these little sensations, so long as we abstain from taking them too seriously. But, alas! as the record abundantly attests, we are but too prone to become permanently "inoculated." We rush off to Germany; spend six weeks, forget our English, and talk "microbe" to our friends.

To any young practitioner about to embark for Germany in search of those minute but attractive creatures, we would say: Don't do it, for the "microbe" is the enemy of practice. If, however, the *feu sacre* is very intense, and refuses to be satisfied, we would recommend our young friend to expend his superfluous vitality in becoming master of modern clinical methods. This can be done as well in New York or Philadephia as abroad.

In doing this there is no danger of curbing originality; indeed, it is doubtful whether there is at the present time a broader or more important field of research than in the domain of scientific therapeutics. The above is especially true of the affections involving the nervous system, where the question of an improved therapy constitutes undoubtedly one of the greatest problems of modern medicine.

Above all things, therefore, let us be thoroughly practical; let us be entirely American, discarding those things which savor, after all, but remotely of usefulness.—*Medical Monthly.*

